# The effect of emotion on morphosyntactic learning in foreign language learners

**DOI:** 10.1371/journal.pone.0207592

**Published:** 2018-11-26

**Authors:** Xinmiao Liu, Xiaodong Xu, Haiyan Wang

**Affiliations:** 1 School of English for Specific Purposes, Beijing Foreign Studies University, Beijing, PRC; 2 School of Foreign Languages and Cultures, Nanjing Normal University, Nanjing, PRC; 3 Language and Brain Science Center, School of Translation Studies, Qufu Normal University, Rizhao, PRC; Nagoya University, JAPAN

## Abstract

Emotions have crucial influence on vocabulary learning and text comprehension. However, whether morphosyntactic learning is influenced by emotional conditions has remained largely unclear. In this study, we investigated how induced positive and negative emotions affect the learning of morphosyntactic rules in a foreign language. It was found that negative emotion increased the accuracy and efficiency of syntactic learning, but had no significant effect on the learning of morphological marking rules. Positive emotion was not found to be significantly associated with learning outcomes. The findings shed light on the effects of affective states on the structural aspects of foreign language learning.

## Introduction

Emotion has a profound impact on our judgments and various cognitive processes [1], coloring how we perceive the outside world. The influence of emotion on psychological function and information processing has been documented in several domains such as perception [2], reasoning and judgment [3], memory storage and retrieval [4]. Language learning is also closely associated with the basic cognitive processes such as perception, categorization, attention and memory [5], making it reasonable to assume that emotion may have influence on language learning as well. According to the Affective Filter Hypothesis [6], the ability to learn a language will be largely constrained by individuals’ affective states. Up until now, although numerous studies have explored the role of emotion in lexical learning [7], [8] and text comprehension [9–11], the question as to whether, and if so, how emotions affect morphosyntactic learning has remained marginally explored.

Morphosyntax is the special property of words which is closely related to syntax with potential morphological, semantic or syntactic effects. Specifically, morphosyntax involves the organization of grammatical elements attached to words, such as the “-s” which is used to signal plural form in English or the “-ga” which is used to mark nominative case in Japanese. Typical morphosyntactic features are gender, case, number and person, which are crucial to the architecture of syntax [12]. While children seem to be able to learn first language morphosyntax with ease, adult foreign language learners often have persistent problems with morphosyntactic features [13], [14], either having difficulty understanding them or making frequent errors. As Chinese is an isolating language, the learning of these features is particularly difficult for Chinese foreign language learners who frequently drop or misuse morphosyntactic markers in writing and speaking. Although prior research has investigated inner-learner variations in morphosyntactic learning such as working memory or cognitive style [15], the role of emotion in morphosyntactic learning has rarely been explored. The aim of this study is to investigate the effect of induced positive and negative emotions on morphosyntactic learning through a semi-artificial grammar task.

### Emotion in language learning

As an essential type of affect, emotion is a complex mental state which is characterized by a certain degree of pleasure or displeasure [16]. A simple way to understand emotion is to consider how it relates to and contrasts with mood, another type of affect with no or a less specific target [16]. Emotions depend on individuals’ general existential states, whereas moods are usually traced as the reactions to specific events localized in time [17]. Emotions may be over in a few seconds or minutes, but moods can last for days or even longer periods [18].

Students are learning foreign language skills in different emotional states on a daily basis. Emotion can offer such a universal context for language learning and processing that its effect can be very pervasive [19]. The relationships between language processing and affective states have been investigated extensively [8, 11, 20, 21]. Prior studies have consistently found that emotions facilitate the memorization and processing of information when its emotional meaning matches the person’s affective states [21], which is known as emotional congruency effect. However, emotional congruency effect only emerges when individuals are engaged in memorization-based activities such as vocabulary learning. Morphosyntactic learning involves a more complex process of categorization, regularization and rule induction, far beyond plain memorization. Students need to extract the morphosyntactic rules of the target language from the input they are exposed to and use the finite number of rules to comprehend or produce an infinite number of novel structures. In this process, it is crucial to classify words from the input into their corresponding grammatical categories, identify regularities underlying the input, and induce the morphosyntactic rules. As morphosyntactic learning requires far more than memorization, emotional congruency effect may not be evident in this process.

The research on the effects of emotion on morphosyntactic learning or processing has been rather limited and whether emotions affect morphosyntactic learning is still not entirely clear. Most previous studies focused on the effects of emotion on the processing of grammatical agreement. However, the findings have been inconsistent. Some studies revealed significant facilitative effects of positive emotion on subject-verb agreement processing while others found that emotion had no significant effect. Vissers et al. [20] studied the effect of induced emotions (positive vs. negative) on the processing of Dutch sentences with subject-verb agreement violations in real time by recording ERPs. A broadly distributed P600 effect was reported for the positive emotional condition and a strong reduction in P600 effect for the negative emotional condition. The authors concluded that the reduced P600 effect reflected reduced syntactic processing in sad emotion conditions, whereas happy emotions may lead to an increase in syntactic processing. This study shows that negative emotions had a detrimental effect on the efficiency of subject-verb agreement processing. However, Van Berkum et al. [11] did not find P600 modulation by emotion in processing subject-verb agreement. Jiménez-Ortega et al. [22] examined the interaction between emotion and noun-adjective number agreement processing. Contrary to previous studies, the study found a detrimental effect of positive emotion on behavioral data. Additionally, clear effects of emotion were found on ERPs. The left anterior negativity (LAN) to agreement violations was present in both negative and positive conditions, but not visible in the neutral condition. No modulation of P600 by emotion was discovered. Whether emotional conditions affect the processing of grammatical agreement is still under debate.

As the studies described above focused on the processing of native language morphosyntax, it is not clear whether the findings can be generalized to foreign language learning. Numerous studies have used artificial grammar tasks to examine the effect of emotion on the learning of a new language [23–26], but the materials adopted were mainly non-meaningful stimuli such as number strings or non-word letter strings which are generated with an underlying grammar. It is not clear whether the findings from these meaningless stimuli can be applied to foreign language learning. The present study intended to use a semi-artificial language to investigate the effect of induced emotions on foreign language learning. The use of a semi-artificial language enables us to examine the learning of nonnative morphosyntactic structures in a natural language. Compared with finite state grammars which are frequently used in artificial grammar learning paradigms, semi-artificial grammars can better preserve the syntactic and lexico-semantic information of natural languages [27]. Thus, the use of semi-artificial languages can provide more convincing evidence regarding the learning and processing of natural languages, and these have been shown to be effective in studies of language learning and processing [15], [28]. Compared with natural languages, semi-artificial languages allow for a more objective assessment of learning outcomes by minimizing the interference of lexico-semantic information on the learning of grammatical rules.

Given that the interactions between emotion and language processing have been reported in many studies [8, 21, 22, 29], it is reasonable to assume that emotional conditions may also influence morphosyntactic learning in a foreign language. If so, foreign language learners could learn to manipulate their emotions to improve their learning performance and language teachers could also use emotion manipulation for classroom teaching. However, it is crucial to empirically evaluate the effects of emotion on foreign language learning before we can use emotion manipulation as a learning or teaching strategy in educational practice.

Learners experiencing positive emotions prefer top-down and global processing while those experiencing negative emotions prefer bottom-up and local processing [30], [31]. As affect assigns value to whatever comes to mind at the specific time, individuals tend to use the affective cues of emotions and moods to make evaluative judgments [32]. In academic situations, positive emotions strengthen the propensity for learners to rely on their previous knowledge or the most accessible knowledge to accomplish learning tasks, fostering a more categorical and global processing style, whereas negative emotions reduce this tendency, resulting in a more and local processing style [33]. As we have discussed previously, morphosyntactic learning requires learners to classify the linguistic inputs into different grammatical categories such as nouns or verbs and then identify the potential connections between these categories. Compared with negative emotions which facilitate a more local processing style, positive emotions which foster more global thinking and a broader scope of attention may place learners in a better position to discover the morphosyntactic rules.

Additionally, previous studies have found that positive emotion facilitates inductive learning while negative emotion enhances deductive learning [34], [35]. Participants feeling negative emotions perform more efficiently in solving deductive reasoning problems as they tend to adopt a more systematic processing strategy, while participants experiencing positive emotions are more efficient in resolving inductive reasoning problems as they can better detect the associations between different items [35]. As morphosyntactic learning is a process of rule induction and categorization, positive-emotion learners may be at a greater advantage than negative-emotion learners. Learners experiencing negative emotions need more time and repetitions during learning, and cannot transfer their knowledge as successfully as their positive-emotion counterparts [36]. Thus, we predicted that positive emotions would facilitate such rule-based learning as morphosyntactic learning, whereas negative emotions would not have a significant effect on this particular learning process.

## Method

### Participants

54 native Chinese speakers participated in this experiment. Participants were recruited from June 24 2016 to July 7 2016. They were all undergraduate students majoring in various fields including journalism, business, law and computer science. They were randomly assigned to two emotion-induction conditions: 27 for positive and 27 for negative emotions. The two experimental groups did not differ significantly in age, *t*(52) = -1.05, *p* = .298, gender ratio, χ^2^ = .11, *p* = .735, education, *t*(52) = -.56, *p* = .578, English language proficiency, *t*(52) = -.48, *p* = .633, or Chinese language proficiency, *t*(52) = -.68, *p* = .499. The participants reported having no reading difficulties and no learning experience with Japanese, the language whose morphosyntactic rules were the target of the present research. This study was performed in accordance with the Declaration of Helsinki and approved by the ethics committee of Beijing Foreign Studies University in China. Participants were recruited via advertisements on social networks. Each participant gave written informed consent immediately prior to the experiment and received monetary compensation after the experiment.

### Materials and design

This study examined the effect of induced emotions (positive vs. negative) on morphosyntactic learning as measured by accuracy and reaction times (RTs). For this purpose, music was used for emotion induction. Participants in the positive-emotion group were asked to listen to *Brandenburg Concerto* and look at forty positive pictures from the Chinese Affective Picture System (CAPS) and those in the negative-emotion group were required to listen to *Alexender Nevsky*: *Russian under the Mongolian Yoke* and look at forty negative pictures from CAPS. A paper-and-pencil version of the Self-Assessment Manikin (SAM) pictorial rating scale was used to acquire valence ratings of the participants after emotion manipulation [37]. Each dimension was measured on a 9-point scale, with higher scores representing more positive valence, higher arousal or stronger dominance. Participants indicated how they felt by marking the corresponding manikin or the space between the manikins. Only the valence dimension was used in the analysis.

In this experiment, a semi-artificial language was used as the target structure which combined Chinese vocabulary with Japanese morphosyntax. It can be used to assess the learning of nonnative morphosyntactic rules without giving participants the extra cognitive load of memorizing new vocabulary. Specifically, this study focused on morphological case marking learning, a crucial dimension of morphosyntactic learning. As morphological marking features are closely related to the linear ordering of sentential constituents, we included both morphological marking and word-order learning in this experiment to shed light on how emotion influences different dimensions of grammar learning. Japanese was chosen as the target language of the current study as it is typologically different from Chinese. Japanese is a language with rich morphological inflections while Chinese is a non-inflected language. The Japanese canonical sentence structure (SOV) and its scrambling structure (OSV) are also different from the typical Chinese syntactic structure (SVO). These typological differences can minimize the transfer effect of native language on foreign language learning and thus allowed for an objective assessment of learning outcomes. Four Japanese sentence structures were used in this experiment including two simple structures (SOV, SIOV) and two complex ones ([SOV]SV, [OSV]SV). The nouns in these sentences were marked for topic (-wa), direct object (-o) or indirect object (-ni). 10 sentences were used for practice, 84 sentences were used for learning and 48 for testing. All sentences with the same word-order structure are of the same length. Sample experimental sentences (a), glossed sentences (b) and their English translation (c) can be found in Table 1.

**Table 1:** Sample sentences used in the study.

Syntactic structure	Sample sentences
SOV	a. 观众は节目を收看了 b. audience-wa program-o watched c. “The audience watched the program.”
SIOV	a. 主人は乞丐に住所を提供了 b. host-wa beggar-ni accommodation-o provided c. “The host provided the beggar accommodation.”
SOVSV	a. 工厂は农田を污染了农民は说 b. factory-wa land-o polluted farmer-wa said c. “The farmer said the factory polluted the land.”
OSVSV	a. 小偷を警察は抓住了路人は说 b. thief-o police-wa caught passer-by-wa said c. “The passer-by said the police caught the thief.”

The experiment consisted of a learning task and a testing task. The learning task was designed to train the participants to learn the target structures and the testing task was devised to evaluate and assess the learning outcomes.

#### Learning task

The stimuli used for the learning task were 84 sentences, including 20 sentences for observation and 64 sentences for grammaticality judgment. In this stimulus set, there were 21 sentences for each structure (13 grammatical and 8 ungrammatical). Sample sentences are presented in Table 2. Before participants performed the learning task, they were informed that they would learn a novel language with Chinese lexicon. Percipients were first instructed to observe 20 sentences (5 for each structure in Table 1) on the computer screen, identify potential regularities and then judge the grammatical acceptability of another 64 sentences within the time limits, with feedbacks (“CORRECT!” or “INCORRECT!”) provided after each response. Correct structures were presented after the feedbacks whether their judgments were correct or incorrect to reinforce the effect of learning. Ungrammatical sentences were designed in such a way that difference types of violations were distributed evenly. All sentences were presented randomly.

**Table 2:** Grammatical and ungrammatical patterns used in the grammaticality judgment task.

Pattern		Sample sentences
**Grammatical**	SOV	a. 妇女は孩子を收养了 b. woman-wa child-o adopted
	SIOV	a. 小王は老板に报告を递交了 b. Xiaowang-wa boss-ni report-o submitted
	SOVSV	a. 市长は农田を视察了农民は说 b. mayor-wa farmland-o inspected peasants-wa said
	OSVSV	a. 医院を病人は离开了医生は说 b. hospital-o patient-wa left doctor-wa said
**Ungrammatical**	#SVO	a. 奶奶は编织了毛衣を b. grandma-wa #knitted sweater-o
	#SIVO	a. 商店は顾客に退还了货款を b. shop-wa customer-ni #returned money-o
	#SVSVO	a. 男孩は说小狗は吃掉了食物を b. #boy-wa said dog-wa ate food-o
	#VOSSV	a. 冰箱を购买了顾客は销售员は说 b. fridge-o #bought customer-wa salesman-wa said
	#-wa	a. 警察小偷を追赶了 b. #police thief-o chased
	#-o	a. 妈妈は面包は烘烤了 b. mom-wa #bread-wa made
	#-ni	a. 服务员は顾客を账单递给了 b. waiter-wa customer-o #bill handed

# ungrammatical morphological marker, word order, or constituent.

#### Testing task

In the testing phase, a grammaticality judgment task was designed to measure learning outcomes. Participants were asked to judge the grammatical acceptability of the given sentences. The testing set consisted of 48 experimental sentences: 24 grammatical and 24 ungrammatical. Ungrammatical sentences either had illicit syntactic order or had licit syntactic order but contained nouns that were missing their morphological markers or were marked incorrectly. The grammatical sentences followed the same four syntactic variations presented during the training phase. The presentation order was randomized for each participant. For this task, participants were required to indicate with a button press (“1” for acceptable and “0” for unacceptable) whether the sentences were grammatically acceptable. Participants were given five seconds to respond and if they failed to respond within the time limit, the next trial started automatically.

#### Procedure

The experiment was conducted over two sessions (a learning session and a testing session). Following the informed consent, participants listened to the music through noise-cancelling headphones which allowed them to be immersed in the music without being distracted by noises. As different participants may have different volume preferences, they were allowed to adjust the volume so that they could hear clearly and comfortably. Affective pictures were presented on the screen. The emotion induction took approximately ten minutes. During the learning session, participants were instructed to rate their emotional valence with the SAM scale before the music induction (Time 1) and immediately after the induction (Time 2). Then they completed the learning task. Participants’ emotion states were measured again immediately before testing (Time 3). In order to assess learning outcomes, we asked participants to perform the testing task after emotion rating. All participants were tested between 9 am to 11 am or between 2 pm to 5 pm, which was counterbalanced between the positive-emotion group and the negative-emotion group.

## Results

### Emotion manipulation

**Fig 1:**
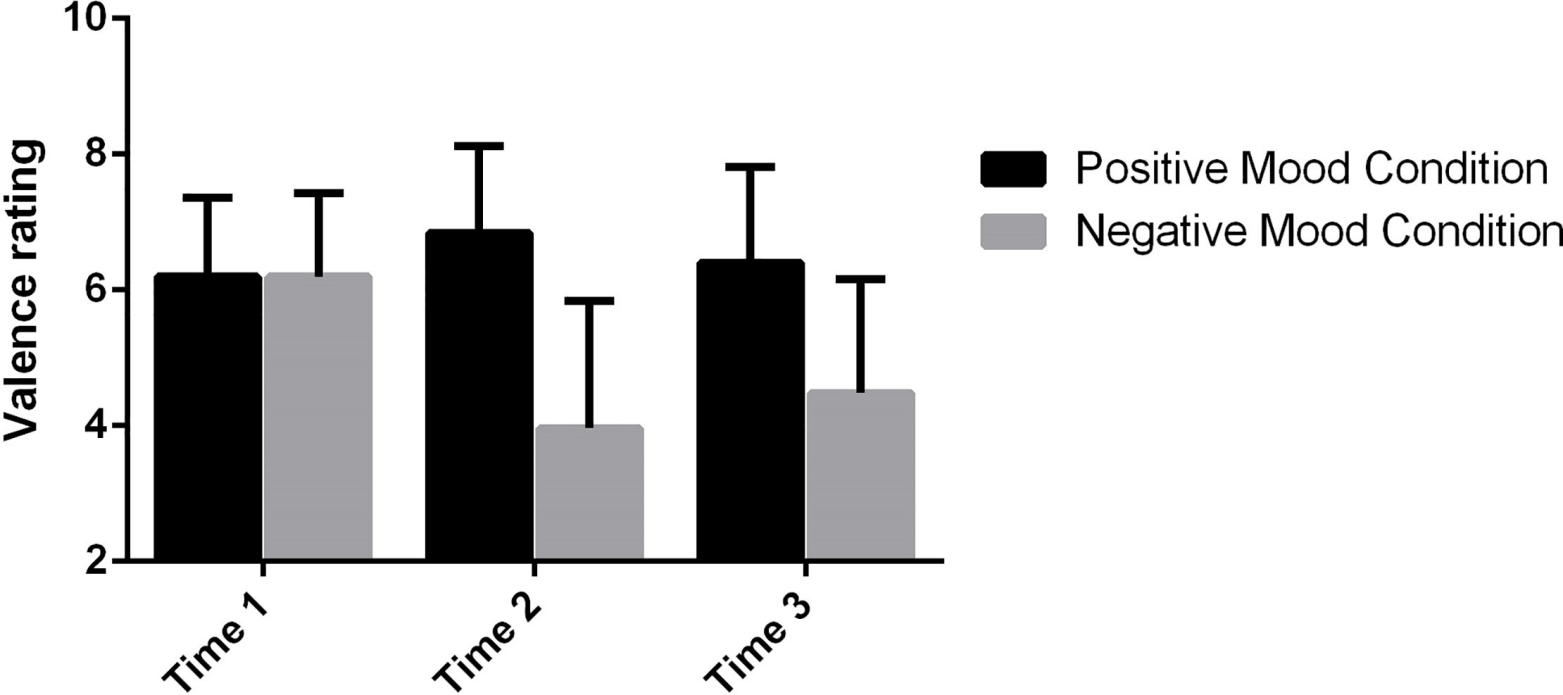
Scores on valence ratings by emotion.

Fig 1 shows the ratings of emotion at three time points in the experiment. The first emotion test was implemented before the emotion induction and the second one was immediately after the induction. To check whether the emotion induction was effective, we performed an ANOVA with time point (first vs. second) as a within-subjects variable and group (negative vs. positive) as a between-subjects variable. The results revealed a significant main effect of time, *F*(1, 53) = 11.93, *p* = .001, a significant main effect of group, *F*(1, 53) = 18.80, *p* < .001, and a significant interaction effect between time and group, *F*(1, 53) = 19.85, *p* < .001. No significant difference between the positive-emotion group and the negative-emotion group was found in emotion ratings before the induction, *t*(52) = .22, *p =* .827. After the induction, the positive-emotion group reported significantly more positive emotions than the negative-emotion group, *t*(52) = 4.56, *p* < .001. Specifically, the induction with the negative-valenced music significantly reduced valence ratings for the negative-emotion group, *t*(52) = 4.15, *p* < .001. The average valence for the negative group was above the neutral level before the induction, *t*(26) = 5.09, *p* < .001, and fell below the neutral level after the induction, *t*(26) = 2.87, *p* = .008, indicating that the emotion induction was successful. The ratings for the positive-emotion group did not increase significantly after the induction with the positive-valenced music, *t*(52) = -1.65, *p* = .105, which shows that the emotion induction was not effective. The average valence for the positive-emotion group was significantly above the neutral level both before the induction, *t*(26) = 5.00, *p* < .001, and after the induction, *t*(26) = 7.85, *p* < .001, indicating that participants were in positive affective states although emotion induction was not effective.

To examine whether the group difference in emotion was sustained during the experiment, we performed an ANOVA with time (second vs. third) as a within-subjects variable and group (negative vs. positive) as a between-subjects variable and found a significant main effect of group, *F*(1, 53) = 29.73, *p* < .001. The rating for the positive-emotion group was significantly higher than that for the negative-emotion group. There was no significant main effect of time, *F*(1, 53) = .30, *p* = .584, or interaction effect between time and group, *F*(1, 53) = 1.01, *p* = .317, which indicates that valence ratings for the two groups did not change significantly from Time 2 to Time 3 and the positive-emotion participants remained happier than their negative-emotion counterparts during this period. T test further confirmed that there was no significant difference in valence ratings between Time 2 and 3 for either the positive-emotion group, *t*(52) = .91, *p* = .365, or the negative-emotion group, *t*(52) = .80, *p* = .426. The results indicated that there was a significant and sustained difference in emotion between the two groups throughout the experiment.

### Effect of emotion on morphosyntactic learning

#### Descriptive statistics

Table 3 presents the descriptive statistics regarding the learning outcomes of both groups. The average accuracy rate for both the positive-emotion group and the negative-emotion group was above 70%, which indicates that both groups were able to achieve significant learning outcomes. The average accuracy rate on the grammaticality judgment test was significantly above chance level, *t*(53) = 18.30, *p* < .001, which showed that participants could discriminate between grammatical and ungrammatical items, as opposed to responding merely in a random way. RTs above or below 2.5 standard deviations from individual means and less than 300 ms were removed, resulting in 3.54% of the data being deleted. Mean RTs were calculated after the elimination of outliners. Questions that were incorrectly answered were excluded from RT analysis. To illustrate whether emotion influenced learning outcomes as well as learning process, we analyzed both the testing data and the learning data.

**Table 3:** Descriptive statistics.

category	structure	Positive emotion		Negative emotion	
		ACC(mean/SD)	RT(mean/SD)	ACC(mean/SD)	RT(mean/SD)
syntax	SOV	83.6% / 0.37	2528.17 / 1018.06	89.5% / 0.30	2272.53 / 928.74
	SIOV	75.9% / 0.42	3213.91/ 1322.71	81.7% / 0.38	3089.96 / 1393.70
	SOVSV	79.3% / 0.40	3065.72 / 1306.87	86.1% / 0.34	2833.83 / 1335.53
	OSVSV	79.0% / 0.40	3086.94 / 1336.55	78.3% / 0.41	3020.00 / 1381.83
	overall	79.4% / 0.40	2924.26 / 1215.58	83.9% / 0.37	2746.40 / 1251.25
case marking	-wa	75.6% / 0.43	3120.83 / 1340.81	75.9% / 0.43	2797.49 / 1234.55
	-o	74.1% / 0.44	2768.04 / 1305.56	76.5% / 0.42	2287.19 / 1080.70
	-ni	76.6% / 0.43	3048.28 / 1220.01	77.2% / 0.42	3027.40 / 1357.26
	overall	75.6% / 0.43	2951.29 / 1263.23	78.1% / 0.41	2639.95 / 1240.67

#### Effect of emotion on morphological marking learning

**Fig 2:**
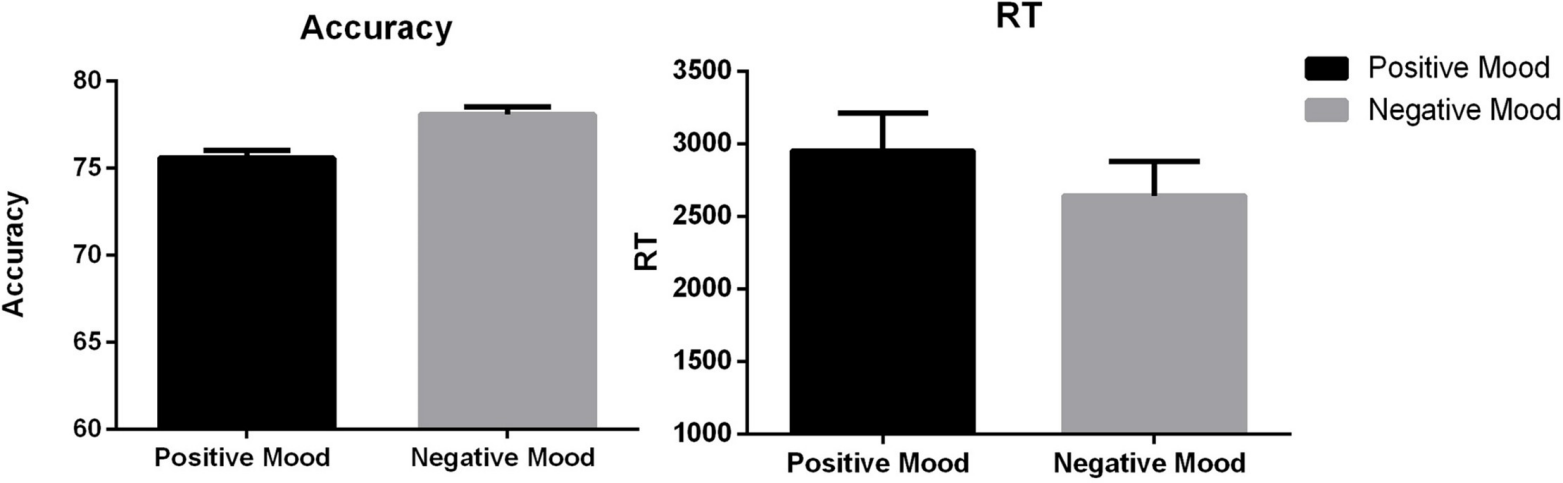
Accuracy and RTs of morphological marking learning.

The accuracy and reaction time of morphological marking learning by emotion conditions are presented in Fig 2. To explore whether there was an effect of emotion on morphological marking learning, we performed an ANOVA test with emotion condition as the fixed factor and overall accuracy in morphological marking learning as the dependent variable. The results indicated that the effect of emotion was not significant, *F*(1, 53) = .55, *p* = .457. The accuracy of morphological marking learning did not differ significantly between the negative-emotion group and the positive-emotion group.

An ANOVA on RTs was also performed with emotion condition as the fixed factor. The results showed that there was a significant effect of emotion on RTs, *F*(1, 53) = 9.77, *p =* .002. RTs for the negative-emotion group were significantly shorter than those for the positive-emotion group, indicating that the negative-emotion learners performed more efficiently than the positive-emotion learners.

We also analyzed whether emotion influenced learners’ learning performance. The results revealed no significant effect of emotion on either accuracy, *F*(1, 53) = .26, *p* = .610, or RTs, *F*(1, 53) = .98, *p* = .322, which indicate that participants’ affective states did not have significant influence on the process of morphological marking learning.

**Fig 3:**
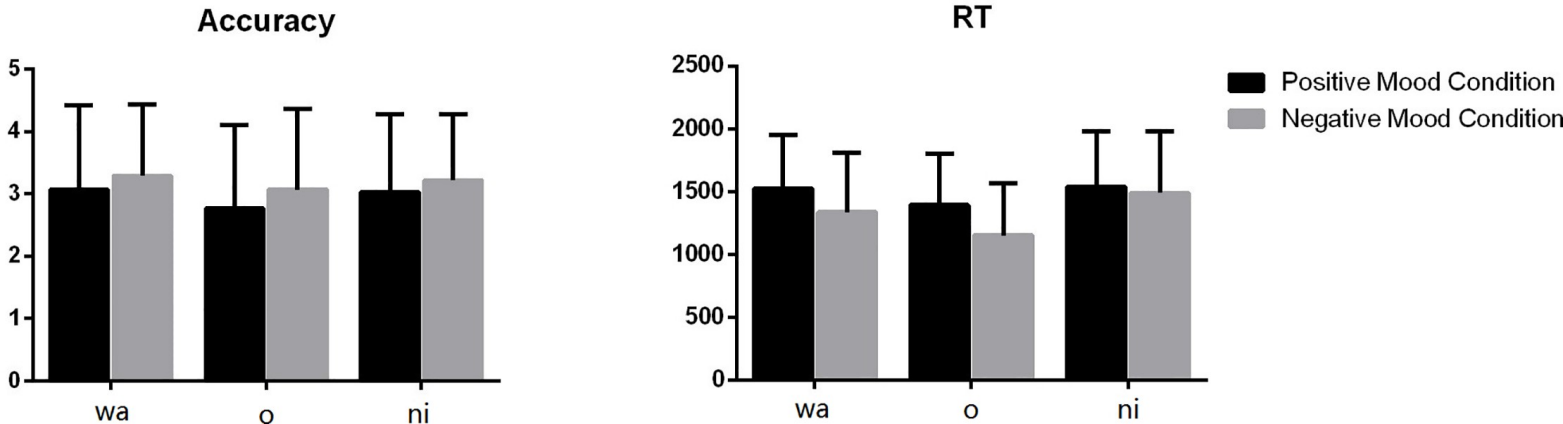
Accuracy and RTs of learning individual morphological markers.

The accuracy and RTs for learning the three morphological marking rules are shown in Fig 3. To examine whether there was an effect of emotion on the learning of particular morphological marking rules, we performed an ANOVA with emotion condition as the fixed factor and accuracy in detecting the violations of each morphological marking rule as the dependent variable. The results revealed no significant effect of emotion on the learning of the topic marker -wa, *F*(1, 53) = .002, *p* = .964, the direct object marker -o, *F*(1, 53) = .13, *p* = .718, and the indirect object marker -ni, *F*(1, 53) = .008, *p* = .931. ANOVA was also performed with emotion condition as the fixed factor and reaction time in detecting three morphological marking violations as the dependent variable. A significant effect of emotion on RTs was found for the direct object marker -o, *F*(1, 53) = 6.52, *p =* .011. The negative-emotion learners performed more quickly in identifying the violations of -o usage than the positive-emotion learners. However, no effect of emotion on RTs was found for the topic marker -wa, *F*(1, 53) = 2.47, *p* = .118, and the indirect object marker -ni, *F*(1, 53) = .01, *p* = .920.

We also examined the effect of emotion on participants’ learning performance. A marginally significant effect of emotion was found on the accuracy of identifying the violations of -o usage, *F*(1, 53) = 3.56, *p* = .061. Learners in the negative affective states had higher accuracy than those in the positive states. Emotion was not found to significantly influence the accuracy of identifying the violation of -wa, *F*(1, 53) = .55, *p* = .459, or -ni, *F*(1, 53) = .50, *p* = .483. The effect of emotion was not significant on RTs for -wa, *F*(1, 53) = .004, *p* = .950, -o, *F*(1, 53) = .33, *p* = .565, or -ni, *F*(1, 53) = .45, *p* = .503.

#### Effect of emotion on syntactic learning

**Fig 4:**
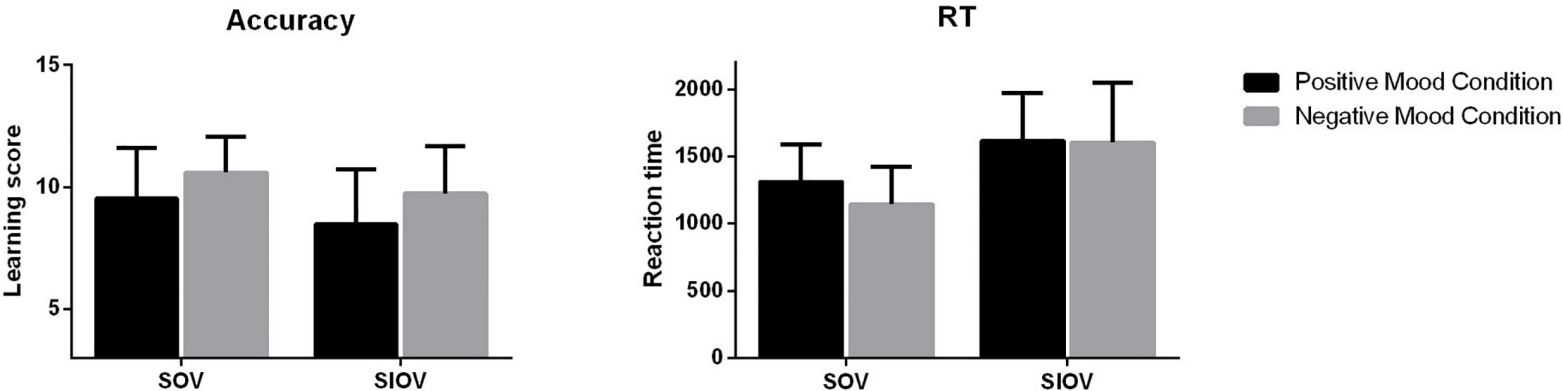
Accuracy and RTs of learning simple syntactic structures.

ANOVA was performed with emotion as the fixed factor and accuracy in learning simple syntactic structures (SOV, SIOV) as the dependent variable. The accuracy and reaction times in learning these two syntactic rules were shown in Fig 4. Results revealed a significant effect of emotion conditions on the accuracy of learning SOV structures, *F*(1, 53) = 4.81, *p* = .028, and a marginally significant effect on the accuracy in learning SIOV structures, *F*(1, 53) = 3.34, *p* = .067. Learners in the negative-emotion group performed more accurately than those in the positive-emotion group in learning SOV structures, *t*(52) = 2.19, *p* = .028, and SIOV structures, *t*(52) = 1.83, *p* = .067.

The results of ANOVA show that there was a significant effect of emotion on RTs in learning SOV structures, *F*(1, 53) = 9.50, *p =* .002. The negative-emotion learners responded to SOV structures significantly faster than the positive-emotion learners. The effect of emotion on the learning of SIOV structures was not significant, *F*(1, 53) = 1.24, *p* = .266.

The analysis with the learning data found no significant effect of emotion on either accuracy, *F*(1, 53) = .009, *p* = .924, or reaction time, *F*(1, 53) = .42, *p* = .518, for SOV learning, which indicates that emotions did not influence the process of SOV learning. There was a marginally significant effect on RTs for SIOV learning, *F*(1, 53) = 3.51, *p* = .061. Negative-emotion learners reacted faster than positive-emotion learners during the learning phase. No significant effect was found on RTs, *F*(1, 53) = .56, *p* = .455.

**Fig 5:**
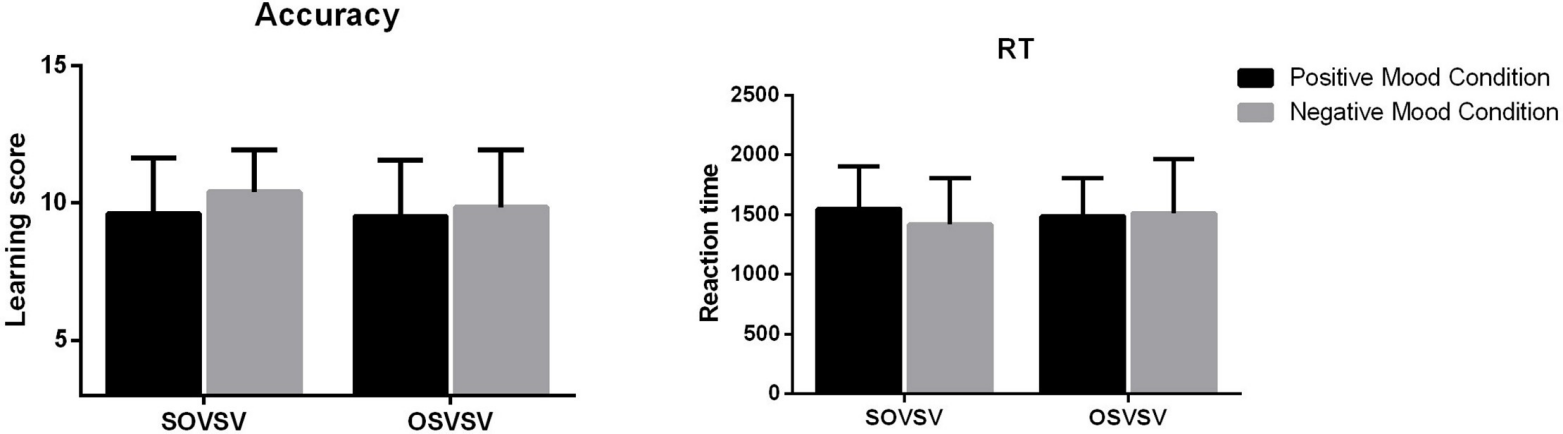
Accuracy and RTs of learning complex syntactic structures.

The accuracy and RTs for learning the two complex sentence structures (SOVSV, OSVSV) are presented in Fig 5. ANOVA was performed with emotion as the fixed factor and accuracy in learning complex syntactic structures as the dependent variable. The results revealed a significant effect of emotion on the accuracy of learning SOVSV structures, *F*(1, 53) = 5.25, *p =* .022. Learners feeling negative emotions were more accurate in responding to questions related to SOVSV structures than learners experiencing positive emotions. The effect of emotion on OSVSV learning was not significant, *F*(1, 53) = .04, *p* = .848, which indicates that the two groups did not differ in the accuracy of their answers to OSVSV-related questions. Negative emotions increased the accuracy of learning SOVSV structures, but the learning of OSVSV structures was not significantly affected by either positive or negative emotions.

ANOVA was performed with emotion as the fixed factor and RTs as the dependent variable. A significant effect of emotion on RTs was found for SOVSV structures, *F*(1, 53) = 4.05, *p =* .044. Learners feeling negative emotion performed significantly faster than their positive-emotion counterparts in responding to questions related to SOVSV structures. ANOVA also found that the effect of emotion on RTs for OSVSV structures was not significant, *F*(1, 53) = .82, *p* = .364, which shows that learners’ emotion conditions had no significant effect on RTs in learning OSVSV structures. Emotions had no significant influence over how fast learners responded to questions related to OSVSV learning.

We also analyzed the effects of emotion on learning performance. The results indicate that for SOVSV learning, there was no significant effect of emotion on accuracy, *F*(1, 53) = .21, *p* = .650, or RTs, *F*(1, 53) = .001, *p* = .988. The effect of emotion on RTs for OSVSV learning was significant, *F*(1, 53) = 9.82, *p* = .002. Learners experiencing negative emotion performed faster in the learning phase. However, there was no significant effect of emotion on accuracy, *F*(1, 53) = .08, *p* = .771.

The analysis above indicates that negative emotion, rather than positive emotion, influenced learning outcomes. Specifically, negative emotion facilitated the learning of SOV and SOVSV structures by improving both accuracy and efficiency. Negative emotion also enhanced the accuracy of SIOV learning. However, the learning of OSVSV structures was largely unaffected by learners’ emotional conditions. Emotion did not affect the accuracy of syntactic learning in the learning phase.

#### Differences in the effect of emotion between morphological marking learning and syntactic learning

To examine whether the effect of emotion differed between syntactic and morphological marking learning, we performed an ANOVA with emotion (positive, negative) and type of violation (syntactic violation, morphological marking violation) as the predictors and accuracy as the dependent variable. The results showed that there was no significant interaction effect between emotion and type of violation, *F*(1, 53) = .09, *p =* .760, indicating that effects of emotion did not differ between syntactic learning and morphological marking learning. The main effect of violation was significant, *F*(1, 53) = 45.08, *p <* .001. The accuracy in detecting morphological marking violations was significantly lower than the accuracy in identifying syntactic violations, *t*(52) = 52.15, *p <* .001. The main effect of emotion was not significant, *F*(1, 53) = .83, *p =* .360.

ANOVA with RTs as the dependent variable revealed a significant main effect of emotion, *F*(1, 53) = 7.89, *p =* .005, and a significant main effect of violation, *F*(1, 53) = 15.97, *p <* .001. The reaction time for identifying morphological marking violations was significantly longer than that for detecting syntactic violations. Learners in the negative-emotion group reacted more quickly to ungrammaticality. The interaction effect between emotion and violation was not significant, *F*(1, 53) = 1.38, *p =* .239, indicating that the effect of emotion on RTs did not differ between syntactic learning and morphological marking learning.

ANOVA with the learning data revealed a significant main effect of violation on accuracy, *F*(1, 53) = 42.26, *p* < .001, showing that syntactic violations were identified more accurately than morphological marking violations. There was no significant main effect of emotion, *F*(1, 53) = .02, *p* = .890, or interaction effect, *F*(1, 53) = .94, *p* = .333. ANOVA also found a significant main effect of emotion on RTs, *F*(1, 53) = 5.32, *p* = .021. The main effect of violation or interaction effect was not significant. The results show that there was no significant difference in the effects of emotion between syntactic and morphological marking learning in the learning phase.

## Discussion

In the present study, we examined the interplay between emotion and foreign language learning. In particular, we focused on the effects of induced positive and negative emotions on morphosyntactic learning. The results were as follows. First, as demonstrated by the analyses of emotion ratings, participants in the negative-emotion group were in a significantly sadder condition after listening to the negative-valenced music, indicating that the emotion manipulation was successful in placing participants in the negative affective states. However, the induction with positive-valenced music was not effective. One possible reason is that the pre-induction valence was positive for both groups in the experiment, which could result in relatively more dramatic changes for the negative-emotion group. Another possible reason is that the positive-valenced music used in this study may not be very effective as the participants with Chinese cultural backgrounds may have difficulty in appreciating the emotional content of western classical music. The results of emotion manipulation indicate that the findings about the effect of positive emotion on learning performance need to be interpreted cautiously. However, as emotion manipulation was effective for the negative-emotion group, this study provides evidence regarding the relationships between negative emotion and language learning. Moreover, as the purpose of this study was to investigate how participants in different emotional states learned language differently, we were more concerned with the ultimate emotional states they were in when they performed the experimental tasks, rather than the process of inducing such emotions. Although the emotion manipulation was not successful for the positive-emotion group, there was a sustained and significant difference in emotional valence between the two groups throughout the experiment. The positive-emotion learners were significantly happier than their negative-emotion counterparts. Therefore, the findings of the current research can still reveal how learners in different emotional conditions learned language differently.

The study revealed a general facilitative effect of negative emotions on morphosyntactic learning. Contrary to our initial prediction that morphosyntactic learning would be enhanced by positive emotions, positive emotions were not found to contribute significantly to learning efficiency or accuracy. One possible explanation is that learners may employ a deductive reasoning strategy in morphosyntactic learning, resulting in a strong reliance on analytical and bottom-up processing. Deductive reasoning is an effective approach to foreign language grammar learning [38]. As participants in this study were informed that they would learn a new grammar and they were asked to search for these rules through observation and grammaticality judgment, they may formulate initial assumptions or premises regarding the potential regularities of the linguistic inputs based on their observation or linguistic experience and use the positive or negative evidence from the subsequent learning process to confirm, revise or abandon the assumptions. As positive emotions promoted the use of inductive strategies, whereas negative emotions enhanced the adoption of deductive strategies, learners in negative emotional conditions may be at an advantage. The findings lend support to the studies which found that adolescent and adult language learners adopted analytical, deductive and declarative learning strategies to learn second language grammar [39], [40].

An alternative explanation is that negative-emotion learners may be more motivated than positive-emotion learners in the learning process. According to Alloy and Abramson’s “sadder but wiser” hypothesis [41], sad individuals can better make judgments about personal behavioral contingencies than those happy individuals. Learners in the negative affective states can accurately assess their performance, whereas those in the positive conditions tend to succumb to cognitive illusions which enable them to overestimate themselves and their environment. Learners in the negative affective states have a more realistic viewpoint about the gap between their actual performance and the desired outcomes and consequently, they are more motivated to improve their performance. This mentality propels them to process information more effortfully and analytically to achieve the goals. Contrarily, individuals experiencing positive emotions are more likely to regard their performance as satisfying and therefore, no further action is considered necessary. In this way, they tend to withdraw efforts and hence effortless and heuristic processing ensues instead of detail-oriented and effortful processing [42–45]. The findings of this study are consistent with previous studies which found that learners feeling positive emotions processed learning materials less carefully and accurately than those in the negative emotional states [46]. The findings are also supported by previous artificial grammar studies which discovered that learners in negative affective states performed better in learning syntactic structures [47].

Additionally, the present study found that emotions selectively influenced the learning of morphosyntactic structures in that negative emotions facilitated the learning of three morphosyntactic structures including SOV, SIOV and SOVSV, but not other structures. The asymmetrical pattern might be attributed to the different complexity or cognitive burden of these linguistic structures. Participants in this study performed less accurately and efficiently in learning the morphological marking rules and OSVSV structures, which indicates that these rules were more difficult to process than other rules. The finding that morphological marking learning was particularly challenging is not surprising because none of the participants in this study had reported any experience of learning languages with overt morphological markers. Thus they may find it difficult to acquire a novel morphosyntactic system, given the relatively limited exposure in the learning phase. In Japanese, OSVSV is regarded as a non-canonical structure which, according to previous studies on Japanese syntax, requires more working memory resources to process than canonical structures such as SOV [48–51]. The results suggested that emotion did not influence the learning of syntactically more complex structures, which might be attributed to the competition between emotional processing and language learning for working memory resources. According to the capacity limitation theory [52], [53], the emotionally-charged information is a cognitive burden and its processing is inherently resource-consuming as it requires the activation of the information network in the brain [52], [53]. Therefore, both positive and negative emotions have deleterious effects on learners’ performance in the tasks which are resource-intensive or require high concentration [54]. In the present study, learners’ performance in learning complex morphosyntactic structures did not differ between the two emotion conditions because the effects of emotion might be inhibited by the greater cognitive load of complex structures.

### Pedagogical implications

The findings bear important pedagogical implications for foreign language teaching. Given the increasing use of multimedia in foreign language teaching, creating a joyful classroom atmosphere through music, pictures or videos is always considered a crucial and sometimes even indispensable part of language teaching in primary schools, secondary schools, universities and language training schools. Many educators are fully convinced that positive emotions can foster stronger interests in language learning among students, reduce their anxiety, keep them more focused on learning tasks and thus improve learning outcome. Teachers who hold this perception tend to spend a considerable amount of time integrating fancy multimedia-based materials such as videos or music into language classrooms. The current study demonstrated that this “the happier, the better” teaching philosophy is not fully supported by scientific evidence and may not necessarily benefit students in every dimension of language learning. Although prior research has revealed an advantage of positive emotions in the memorization of emotionally positive words [55], no such advantage has been found in the learning of morphosyntax. The indiscriminate use of emotion induction may lead to ineffective teaching. Language teachers should be mindful of the use of emotion-induction materials and avoid using them indiscriminately in all learning tasks. For effective teaching, language teachers should realize that the use of emotion-induction materials needs to be tailored to maximize language learning success. For the learning tasks which require more focus of attention and analytical thinking, the use of negative-valenced learning materials might be an effective teaching strategy. For foreign language learners, the finding that negative emotions enhanced grammar learning might imply that learners should place themselves in a somewhat negative affective state before performing the tasks which require analytical thinking and attention to details.

### Limitations

As with almost all studies, this research has its limitations. First, this study only tested the immediate learning performance and the deferred performance was neglected. It is not clear whether the observed effect of emotion was sustained. The long-term effect of emotion on learning should have been investigated to further our understanding of the relationships between affective states and language learning. Second, the study merely focused on the effects of emotion on language comprehension and did not provide much information as to how language production may be associated with learners’ emotional conditions. Further research is needed to examine the effects of emotion on the different stages of language production between developing a concept, and translating that concept into linguistic forms. The study highlights the importance of evaluating language learning under different emotional conditions. As such, future research should also incorporate into their designs other areas, such as phonology or semantics. Third, as a semi-artificial language task was used in the present study, it might not be clear whether the findings can be generalized to natural language learning. However, given that a considerable number of studies have adopted artificial or semi-artificial language paradigms to investigate second language learning or processing [56–59] and first language acquisition [60], it is convinced that the findings from the current study can offer inspirations for future research on natural language learning.

## Conclusions

This study examined the effects of induced negative and positive emotions on morphosyntactic learning. The research design allowed us to draw conclusions about the relationships between learners’ affective states and their performance in learning a nonnative language. This study found that negative emotions facilitated morphosyntactic learning in foreign language learners. Given that cognition has always been prioritized over emotion in the studies of foreign language acquisition [61], the current study provides crucial evidence for the inseparability of cognition and emotion in the learning process. The findings also have important implications for the theoretical understanding of morphosyntactic learning, suggesting that there might be deductive aspects of morphosyntactic learning. Hopefully future research in this area can continue to expand our knowledge of the complicated interplay between emotion and foreign language learning.

## Supporting information

S1 Dataset Dataset for morphosyntactic learning in foreign language learners. (RAR)
